# Phytochemical Investigation of Sumac (*Rhus coriaria* L.) Fruits from Different Sicilian Accessions

**DOI:** 10.3390/foods12234359

**Published:** 2023-12-02

**Authors:** Eugenia Mazzara, Arianna Caprodossi, Ahmed M. Mustafa, Filippo Maggi, Giovanni Caprioli

**Affiliations:** Chemistry Interdisciplinary Project (CHIP), School of Pharmacy, University of Camerino, Via Madonna delle Carceri, 62032 Camerino, Italy; eugenia.mazzara@unicam.it (E.M.); arianna.caprodossi@studenti.unicam.it (A.C.); ahmed.mustafa@unicam.it (A.M.M.); giovanni.caprioli@unicam.it (G.C.)

**Keywords:** sumac, Sicilian accessions, water extracts, HPLC-MS/MS, antioxidants, food supplements, nutraceuticals

## Abstract

Sumac, *Rhus coriaria* L., is employed as a natural preservative in the food sector, due to its rich content of antioxidant compounds, including hydrolysable tannins, phenolic acids, anthocyanins, and flavonoids. In this work, the phytochemical characterization of sumac fruits from five Sicilian accessions was performed to evaluate their potential as a food preservative for nutraceutical exploitation. Spectrophotometric tests and HPLC-MS/MS analyses were conducted to assess and compare the antioxidant power of the water extracts produced with the five sumac accessions. Principal component analysis was also carried out to better visualize the obtained results. Flavonoids and phenolic acids, namely isoquercitrin (20,342.82 mg/kg dry extract) and gallic acid (197,489.19 mg/kg dry extract), were more abundant in fruits from the population of San Biagio Platani, while the one from Giarratana was characterized by a higher content of anthocyanins such as cyanidin-3-glucoside (20,889.81 mg/kg dry extract). These two populations can be recognized as the most suitable settings for the implementation of sumac cultivation and the development of sumac-based products, especially for food and nutraceutical purposes.

## 1. Introduction

*Rhus coriaria* L. (Anacardiaceae), commonly known as sumac, is a 1–3 m high shrub or small tree possessing reddish or dark-brown, spherical, and fluffy drupe fruits, with dark-purplish glandular hairs, arranged in clusters, deriving from dense panicles. It can grow widely (between hedges, along roads, and up to 600–700 m a.s.l.), and it has been cultivated for several centuries in poor soils [1]. This species is diffused mainly in temperate and tropical regions, especially in Mediterranean, Middle Eastern, and Western Asian shores. It is employed as a flavoring spice and plant medicine, especially in Iran, Turkey, Palestine, Israel, and Jordan [2]. In the past, sumac has been used as a natural and traditional remedy, particularly in the treatment of diarrhea, liver disease [3], ulcer [4], hemorrhoids, animal bites, pain [5], dysentery, diuresis, hemorrhage, ophthalmia, conjunctivitis, and as a stomach tonic. Its use has also been indicated for cholesterol reduction, in the treatment of sore throat, and as an abortifacient [6]. Several biological properties have been reported for sumac, including the antibacterial [7], hepatoprotective [8], antifungal [9], antioxidant, anti-inflammatory [10], DNA-protective [11], anti-ischemic, vasorelaxant [12], antidiabetic [13], anticancer [14], and antinociceptive [15] effects. From an industrial point of view, *R. coriaria* is employed in the textile field as a tanning agent, especially for leather, and as a natural dye, with a high fixation, retention, and fungal resistance properties, being effective against wood decay [16]. Notably, sumac aqueous extract was shown to improve the quality of a Turkish fermented sausage [17] and to enhance the shelf life of rabbit meat [18], while the ethanolic extract possessed antimicrobial effects in minced meat [19], suggesting an important role of sumac as a natural preservative in the food industry. The numerous above-mentioned pharmacological activities and applications of sumac can be correlated with the presence of more than 200 biomolecules in the plant. Among them, the most important ones are hydrolysable tannins, phenolic acids, anthocyanins, and flavonol glycosides [20,21,22,23]. Recently, innovative extraction techniques based on deep eutectic solvents, ultrasounds, and microwaves [24] have been introduced for sumac to extract the aforementioned bioactive compounds. In this work, a phytochemical analysis of sumac fruits from different Sicilian populations was carried out to evaluate their phytonutrients as food preservatives and for nutraceutical purposes. More specifically, after a preliminary extraction optimization aimed at identifying the best extraction conditions, the antioxidant capacity and the content of bioactive compounds, especially phenolic acids and anthocyanins, were investigated in the fruit extracts from five Sicilian sumac populations, by means of spectrophotometric assays and HPLC-MS/MS analyses. The final objective of this study was to identify the most suitable locations in Sicily for implementing sumac cultivation and developing sumac-based products, especially for food and nutraceutical applications.

## 2. Materials and Methods

### 2.1. Plant Material

Sumac fruits, harvested in September 2021, were provided by the company Redess S.r.l. (https://www.redess.it), sited in Termini Imerese (PA). Initially, they were used, after being dried, to preliminarily find the best extraction conditions. Secondly, five batches of fruits belonging to different Sicilian populations, namely Termini Imerese (37°59′4″ N, 13°41′47″ E, 77 m a.s.l.), Castronovo di Sicilia (37°40′44″ N, 13°36′12″ E, 677 m a.s.l.), San Biagio Platani (37°30′33″ N, 13°31′42″ E, 416 m a.s.l.), Alessandria della Rocca (37°34′3″ N, 13°27′15″ E, 533 m a.s.l.), and Giarratana (37°2′58″ N, 14°47′42″ E, 520 m a.s.l.), were provided by the company, and after drying (r.t.), transported to the University of Camerino where they were ground and stored at room temperature before use.

### 2.2. Preparation of Extracts

Four kinds of extracts (water extracts at 25, 40, and 100 °C and water/ethanol (1:1) extract) were prepared using sumac fruits [25]. In the case of water extractions, 5 g of fruits were weighed, and 35 mL of distilled water were added. After homogenization, the mixture was extracted in a water bath for 1.5 h at 25, 40, and 100 °C, respectively. For the water/ethanol extraction, 5 g of fruits were added to 35 mL of a 1:1 mixture of the two solvents, homogenized, and extracted in a water bath for 1.5 h at 45 °C. All the obtained extracts were then filtered and kept at − 20 °C. The liquid extracts were freeze-dried using liquid nitrogen. The most effective extraction conditions were selected and then applied on fruit samples collected from five different Sicilian sites. The extraction yield was calculated (% *w*/*w*), and the lyophilized extracts were used for further analyses.

### 2.3. Spectrophotometric Assays

For performing the tests, 10 mg of water extracts were dissolved in 5 mL of water, while 10 mg of 1:1 water/ethanol extract were solubilized in 5 mL of the same aqueous/ethanolic mixture. For this reason, in all cases, an ultrasonic bath from FALC, Treviglio, Italy, was employed with a frequency of 59 KHz for 1 h at 25 °C [26].

#### 2.3.1. DPPH Radical Scavenging Activity

The 2,2-diphenyl-1-picrylhydrazyl (DPPH) free radical scavenging activity of fruit extracts was determined according to the method of Mustafa et al. (2016) [27], with some modifications. In particular, 10 µL of extract solution were diluted with 990 µL of water. Then, 9 mL of EtOH solution of DPPH (0.1 mM) were added. After 30 min of incubation at room temperature in the dark, the disappearance of DPPH was evaluated spectrophotometrically at 517 nm. Trolox was employed as the reference antioxidant, and the results were calculated as mg Trolox equivalents (TE)/g dry extract (DE). Experiments were performed in triplicate.

#### 2.3.2. Total Phenolic Content

Total phenolic content (TPC) was performed through the Folin–Ciocalteu method [27], with few modifications. Specifically, 50 µL of extracts solution were diluted in 450 µL of water (dilution 1:10). Then, 2.5 mL of Folin–Ciocalteu in water and 7 mL of a 7.5% Na_2_CO_3_ aqueous solution were added. The reaction mixture was kept in the dark at room temperature for 2 h, and the absorbance was registered at 765 nm by using the Cary 8454 UV-Vis spectrophotometer (Agilent Technologies, Woburn, MA, USA). The quantification of phenolic compounds was made by using gallic acid standard and its calibration curve. TPC was expressed as gallic acid equivalents per g of dry extract (mg GAE/g DE). The results were calculated as the mean of three experiments. 

#### 2.3.3. Total Flavonoid Content

The total flavonoid content (TFC) was carried out spectrophotometrically [28], with the same above-mentioned instrument. In detail, 0.5 mL of the extract’s solution, 0.15 mL of 0.5 M NaNO_2_, 3.2 mL of 30% MeOH (*v*/*v*), and 0.15 mL of 0.3 M AlCl_3_·6H_2_O were mixed. Then, 1 mL of 1 M NaOH was added after 5 min. The reaction mixture was shaken, stored in the dark for 30 min, and the absorbance was recorded against a blank reagent at 506 nm. The standard calibration curve for TFC was built using quercetin standard solutions, and the TFC was calculated as mg of quercetin equivalents per g of dry extract (mg QE/g DE). Analyses were performed in triplicate.

#### 2.3.4. Total Anthocyanin Content

The total anthocyanin content (TAC) was quantified according to the differential pH method [29]. A 1:10 dilution of the extracts was performed by using 0.025 mol/L KCl buffer at pH = 1 and 0.4 mol/L CH_3_COONa buffer at pH = 4.5. The mixtures were kept in the dark at room temperature for 15 min, so their absorbance was read at 510 and 700 nm. The TAC was estimated through the following formula:

TAC = [(A_510nm_ − A_700nm_) pH_1.0_ − (A_510nm_ − A_700nm_) pH_4.5_] MW × TV × DF × 1000/(ε × L × SW), (1)

where A is the absorbance, MW is the molecular weight of cyanidin-3-glucoside, which corresponds to 449.2 g/mol^−1^, TV is the total extract volume, DF represents the dilution factor, ε is the extinction coefficient, equal to 22,400 L/(mol × cm), L is the length of the cuvette of 1 cm, and SW represents the weight of the sample or starting material. The results were expressed as mg of cyanidin-3-glucoside equivalents per g of dry extract (mg CGE/g DE). Analyses were performed in triplicate.

### 2.4. HPLC-MS/MS Analysis

The HPLC-MS/MS investigation was performed using an Agilent 1290 Infinity series and a Triple Quadrupole 6420 from Agilent Technology (Santa Clara, CA, USA), equipped with an electrospray ionization (ESI) source, operating in negative and positive ionization modes [30]. The MS/MS parameters of each analyte were optimized in flow injection analysis (FIA) (1 μL of a 10 mg L^−1^ individual standard solution), by using the Mass Hunter Optimizer Software (Agilent, Santa Clara, CA, USA). The separation of compounds of interest was conducted on a Synergi Polar–RP C18 analytical column (250 mm × 4.6 mm, 4 µm) from Phenomenex (Chesire, UK). The column was preceded by a Polar RP security guard cartridge (4 mm × 3 mm). The mobile phase was a mixture of (A) H_2_O and (B) MeOH, both with HCOOH 0.1%, at a flow rate of 0.8 mL min^−1^ in gradient elution mode. The composition of the mobile phase changed as follows: 0–1 min, isocratic condition, 20% B; 1–25 min, 20–85% B; 25–26 min, isocratic condition, 85% B; 26–32 min, 85–20% B. All the solvents and solutions were filtered through a 0.2 μm polyamide filter from Sartorius Stedim (Goettingen, Germany). The injection volume was 2 μL. The temperature of the column was 30 °C, and the temperature of the drying gas in the ionization source was 350 °C. The gas flow was 12 L min^−1^, the nebulizer pressure was 55 psi, and the capillary voltage was 4000 V. The detection was performed in dynamic-multiple reaction monitoring (dynamic MRM) mode, and the dynamic MRM peak areas were integrated for quantification. Cyanidin-3-glucoside chloride, delphinidin-3,5-diglucoside chloride, delphinidin-3-galactoside chloride, petunidin-3-glucoside chloride, malvidin-3-galactoside chloride, quercetin-3-glucoside, and kaempferol-3-glucoside were purchased from PhytoLab (Vestenbergsgreuth, Germany). The other 29 analytical standards were supplied by Sigma-Aldrich (Milan, Italy). The most abundant product ion was used for quantitation, and the others for qualitative analysis. The specific time window for each compound (Δ retention time) was set at 2 min. For HPLC analysis, the dried extracts were dissolved in methanol/water (1:1) (10 mg/mL) by sonication (ultrasonic bath FALC, Treviglio, Italy), at a frequency of 59 KHz for 30 min at 25 °C, then centrifuged for 10 min at 13,000 rpm. Prior to being injected into the HPLC–MS/MS system, the solutions were filtered using a 0.2 µm syringeless filter [26]. For each extract, analyses were performed in triplicate.

The analytical method was validated in terms of linearity, limits of detection (LODs), limits of quantification (LOQs), repeatability, and specificity. Calibration curves were constructed by injecting standard mixture solutions at the seven concentrations of 0.005, 0.01, 0.05, 0.1, 0.5, 1, and 5 mg/L, and the phenolic compounds showed good linearity (R^2^ ≥ 0.9958). The LODs ranged from 0.0004 to 0.0033 mg/L, while the LOQs were defined in the range of 0.0012 to 0.01 mg/L, indicating very good sensitivity. The intraday precision of the HPLC-MS/MS method was evaluated by injecting analytical standard dissolved in solvents at the same concentration, i.e., 1 mg/L thrice. The interaday precision was evaluated by injecting the same concentration of analytical standard dissolved in solvents for three different consecutive days. All of the precision measurements were expressed as relative standard deviations (RSDs). The method revealed a very good precision with inter- and intraday variations where RSD (%) ranged from 0.21 to 3.23 and 0.11 to 2.88, respectively. The method specificity was evaluated by measuring the stability of retention time for three times over a period of 3 days and expressed by RSDs %, which were in all cases ≤ 1.12%.

### 2.5. Principal Component Analysis

Principal component analysis (PCA) was conducted through the STATISTICA software v. 7.1 (Stat Soft Italia S.r.l., Vigonza, Italy) by using the HPLC-MS/MS results (Section 3.3.3) for the composition of the extracts obtained from the five Sicilian sumac populations organized in a covariance matrix. The score and loading plots were built to display the clustering of samples and contribution to variance of different compounds, respectively.

## 3. Results

### 3.1. Antioxidant Activity of the Preliminary Extracts

**Figure 1:**
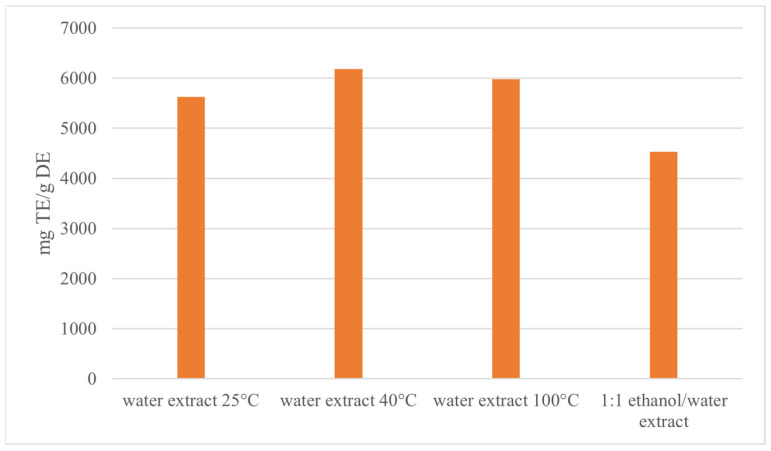
Antioxidant activity results for the sumac fruits extracts expressed as mg TE/g DE.

The antioxidant activity assay was conducted spectrophotometrically through the DPPH method on the sumac fruits extracts obtained via water extraction at 25, 40, and 100 °C and using a 1:1 water/ethanol mixture. As a result, the water extract at 40 °C showed the best radical scavenging activity (Figure 1). On this basis, in order to confirm the greater performance of this extract with respect to the others in terms of content of bioactive compounds endowed with antioxidant activity, all four extracts underwent HPLC-MS/MS characterization.

### 3.2. HPLC-MS/MS Characterization of the Preliminary Extracts

As represented in Table 1, the water extract at 40 °C was confirmed to be the most valuable one in terms of concentration and number of bioactive constituents identified and quantified through HPLC-MS/MS analysis. The most abundant compound in this extract was gallic acid, followed by the anthocyanin cyanidin-3-glucoside, the flavonol glycosides isoquercitrin, quercitrin, and hyperoside, and the phenolic acid ellagic acid. In the other extracts, the levels of such antioxidant components were much lower than those found in the water extract at 40 °C. Hence, the latter conditions were selected and applied for the sumac fruits deriving from the five Sicilian populations.

### 3.3. Analysis on the Five Sumac Fruit Samples from Sicily

#### 3.3.1. Yield of the Extracts Made by the Five Sumac Accessions

The detected yields were similar for the five water extracts prepared at 40 °C using the above-mentioned Sicilian sumac populations. The highest yields were found for Castronovo di Sicilia fruit extract and, secondly, for Termini Imerese fruit extract, while the lowest value was registered for San Biagio Platani fruit extract (Table 2).

#### 3.3.2. Spectrophotometric Assays of the Sumac Fruit Extracts from the Five Sicilian Accessions

Regarding TAC, the best results were obtained for Giarratana fruit extract (24.07 mg CGE/g DE), while the most promising extract in terms of TPC (473.08 mg GAE/g DE), TFC (55.56 mg QE/g DE), and DPPH radical scavenging activity (4111.11 mg TE/g DE) was that from San Biagio Platani fruits (Table 3). 

#### 3.3.3. HPLC-MS/MS Analysis of the Sumac Fruit Extracts from the Five Sicilian Accessions

The major components detected in sumac water extracts from the five Sicilian collection sites were, among flavonoids, isoquercitrin, quercitrin, kaempferol 3-glucoside, rutin, and quercetin. Except for rutin, which was found in the highest content in Termini Imerese sumac extract, the other main flavonoids were more concentrated in San Biagio Platani sumac extract. The main quantified anthocyanins were cyanidin 3-glucoside and delphinidin-3,5-diglucoside. Except for delphinidin-3,5-diglucoside, which was detected in higher amounts in San Biagio Platani sumac extract, all the other anthocyanins were prevalent in Giarratana sumac extract (Table 4). Gallic, ellagic, *p*-hydroxy benzoic, and *p*-coumaric acids were the most abundant phenolic acids, and, again, San Biagio Platani sumac extract presented the most significant levels of these compounds. 

#### 3.3.4. Principal Component Analysis

**Figure 2:**
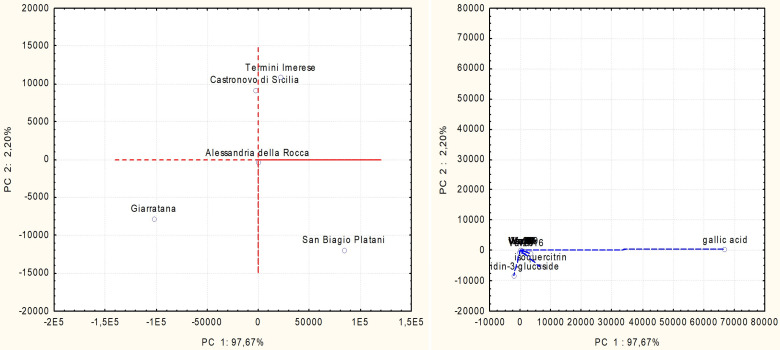
PCA score and loading plots depicting sumac accessions and constituents of their extracts.

The PCA (Figure 2) carried out on the sumac accessions and phenolic compounds showed data variability of 97.67% on the first principal component (PC 1) and 2.20% on the second one (PC 2). 

The variance was determined almost exclusively by gallic acid on PC1, while cyanidin-3-glucoside and isoquercitrin gave a negligible contribution along PC2. San Biagio Platani accession extract was distinguished for the highest content of gallic acid and isoquercitrin, while cyanidin-3-glucoside mostly characterized the extract obtained from the Giarratana population.

#### 3.3.5. Correlation between Antioxidant Capacity and TPC, TFC, and TAC Determined by Spectrophotometric Assays

**Figure 3:**
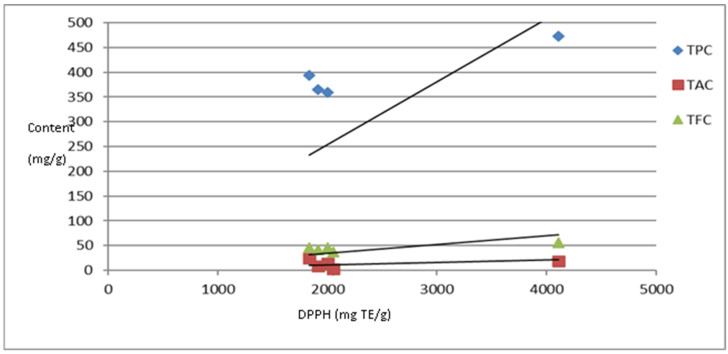
Regression analysis of AOC and TPC, TAC, and TFC in sumac samples.

The DPPH assay was used to evaluate the antioxidant capacity (AOC) of the sumac samples, and the obtained values were statistically compared with the amounts of TPC, TFC, and TAC determined in each sample using Pearson correlation test. The relationships between phytochemical contents and AOC are presented in Figure 3. AOC showed the highest correlation with TPC (r = 0.93, *p* < 0.05), while it demonstrated a non-significant relationship with TFC and TAC as *p* > 0.05.

## 4. Discussion

The water extraction at 40 °C was confirmed to be the most suitable one in terms of radical scavenging activity and content of bioactive constituents identified and quantified through HPLC-MS/MS analysis.

The differences among sumac extracts of various provenance in terms of spectrophotometric analyses results can be related to the variability in the geographical positions, with diverse climate and soil conditions. In fact, in another work exploring the variations in fatty acids content and antioxidant effects among several Iranian populations of sumac fruits, two accessions from temperate mountainous areas in a non-stressful condition were shown to possess the highest polyphenol content. However, simple correlation analysis revealed a negative correlation between TPC and annual mean temperature [31]. In general, multiple factors, including gene expression patterns, habitat parameters (sunlight, soil, and temperature), and pre- and post-harvesting conditions, can influence plant total phenol production [32].

Overall, Termini Imerese fruit extract presented poorer results than the other extracts in terms of TPC, TFC, and TAC, possibly due to the proximity of this area to the sea, which differentiates it from the other hinterland locations. Notably, the TPC values found in this study were higher than those previously reported for sumac aqueous ethanol extract (159.32 mg GAE/g DE) [33] and aqueous extract (55.16 mg GAE/g DE) [34].

HPLC-MS/MS findings confirmed the results obtained by the spectrophotometric assays, demonstrating that the investigated sumac accessions possessed an interesting profile in terms of antioxidant molecules, with San Biagio Platani and Giarratana standing out for phenolic constituents and anthocyanins content, respectively.

Generally, the outcomes of this work were in line with those found in the literature, and the variability in target compound levels may be due to the applied extraction methods employing different plant parts and solvents [25]. Indeed, phenolics and anthocyanins were successfully extracted, especially from sumac leaves and fruits, by using organic solvents, also in combination with each other, such as methanol, ethyl acetate, and ethanol [20,21,22,35,36]. Nevertheless, water appeared to be a proper extracting agent, and, in particular, it was indicated as the best solvent for gallotannins recovery [23,37]. Moreover, sumac fruit extracts prepared by solvent immersion have been reported to increase the shelf life of soybean oil more efficiently than ultrasound- and microwave-assisted extraction due to a higher release of antioxidants in the solvent [38]. 

In this study, a sustainable one-step extraction process employing only water was applied to produce safe sumac extracts, with the specific aim to identify the preferable Sicilian sumac populations based on antioxidant features to be potentially used in the food industry.

In a previous work, sumac fruits from four Sicilian accessions, including Castronovo di Sicilia, were compared for the volatile composition [39]; to the best of our knowledge, a similar approach aimed at investigating the antioxidant profile of the valuable ‘Sommacco siciliano’ of diverse geographical origin represents a novelty in the literature framework. 

## 5. Conclusions

In this study, phenolic acids, anthocyanins, and flavonol glycosides were extracted from several Sicilian accessions of sumac fruits, revealing their suitability for the development of natural preservatives for the food sector. A preliminary extraction optimization was carried out, leading to identification of the water extraction at 40 °C as the best process to obtain enriched extracts, especially in gallic acid, cyanidin-3-glucoside, isoquercitrin, quercitrin, hyperoside, and ellagic acid. Subsequently, a phytochemical characterization of the water extracts from five Sicilian sumac populations was performed via spectrophotometric tests and HPLC-MS/MS analyses. Flavonol glycosides and phenolic acids were more abundant in the extract from San Biagio Platani, especially isoquercitrin and gallic acid, while the extract from Giarratana showed a higher content of anthocyanins, particularly cyanidin-3-glucoside. In conclusion, San Biagio Platani and Giarratana can be considered as the most suitable locations where sumac cultivation should be supported and boosted for the development of sumac-based products, especially for food and nutraceutical applications.

## Figures and Tables

**Table 1:** Content (mg/kg DE) of bioactive compounds detected by HPLC-MS/MS analysis of the sumac preliminary extracts; standard deviations were in all cases ≤ 9.45.

Compound	Sample			
	Water Extract 25 °C	Water Extract 40 °C	Water Extract 100 °C	1:1 Ethanol/Water Extract
	mg/kg DE			
Gallic acid	728.28	34,693.70	2275.21	20.98
Neochlorogenic acid	1.53	6.05	1.01	0.44
Catechin	n.d. ^1^	47.14	n.d.	n.d.
Chlorogenic acid	4.88	5.41	0.66	0.98
*p*-Hydroxy benzoic acid	n.d.	152.87	29.50	n.d.
Epicatechin	n.d.	1.53	n.d.	n.d.
*m*-Hydroxy benzoic acid	n.d.	256.32	32.54	n.d.
Caffeic acid	n.d.	20.59	n.d.	n.d.
Vanillic acid	n.d.	36.68	n.d.	n.d.
Syringic acid	n.d.	12.05	n.d.	n.d.
*p*-Coumaric acid	0.53	54.90	1.84	n.d.
Ferulic acid	n.d.	13.86	n.d.	n.d.
3,5-Dicaffeoylquinic acid	0.45	n.d.	3.50	n.d.
Rutin	0.16	105.23	1.32	n.d.
Isoquercitrin	15.44	3825.78	81.45	0.72
Delphinidin-3,5-diglucoside	2.40	743.75	13.75	0.22
Phloridzin	0.18	53.17	0.83	n.d.
Quercitrin	7.08	2039.73	40.79	0.38
Myricetin	n.d.	4.46	n.d.	n.d.
Naringin	n.d.	118.70	n.d.	n.d.
Kaempferol-3-glucoside	0.93	199.02	4.50	n.d.
Ellagic acid	4.65	1269.15	19.05	n.d.
Quercetin	n.d.	10.56	n.d.	n.d.
Phloretin	n.d.	0.03	n.d.	n.d.
Isorhamnetin	n.d.	0.04	0.04	n.d.
Delphinidin-3-galactoside	1.30	219.70	5.15	n.d.
Cyanidin-3-glucoside	49.12	6088.67	193.91	2.69
Petunidin-3-glucoside	n.d.	19.62	0.58	n.d.
Pelargonidin-3-glucoside	0.45	62.83	1.81	n.d.
Hyperoside	8.25	1406.91	35.36	n.d.
Hesperidin	n.d.	7.02	n.d.	n.d.
*trans*-Cinnamic acid	n.d.	0.96	n.d.	n.d.
Kaempferol	n.d.	7.59	n.d.	n.d.
Total content	825.63	51,484.00	2742.81	26.41

^1^ n.d., not detected.

**Table 2:** Yield values of the five Sicilian sumac water extracts obtained at 40 °C.

Sample	Yield (% *w*/*w*)
Termini Imerese	11.6
Castronovo di Sicilia	11.8
San Biagio Platani	10.2
Alessandria della Rocca	10.8
Giarratana	11.4

**Table 3:** Results of the spectrophotometric tests of sumac extracts from the five Sicilian locations.

Sample (Extract)	TPC (mg GAE/g DE)	TFC (mg QE/g DE)	TAC (mg CGE/g DE)	DPPH (mg TE/g DE)
Termini Imerese	354.81 ± 3.45	38.06 ± 3.10	2.50 ± 3.35	2055.56 ± 9.47
Castronovo di Sicilia	365.38 ± 4.24	41.67 ± 9.43	9.18 ± 2.78	1916.67 ± 3.69
San Biagio Platani	473.08 ± 2.87	55.56 ± 7.32	18.73 ± 4.58	4111.11 ± 7.64
Alessandria della Rocca	358.65 ± 1.90	46.67 ± 1.68	15.01 ± 3.23	2000.00 ± 7.86
Giarratana	393.27 ± 1.73	46.11 ± 5.21	24.07 ± 1.99	1833.33 ± 4.29

**Table 4:** Content (mg/kg DE) of bioactive compounds in sumac samples analyzed by HPLC-MS/MS; relative standard deviations % were in all cases ≤ 7.32.

Compound			Sample		
	Castronovo Di Sicilia Extract	San Biagio Platani Extract	Giarratana Extract	Termini Imerese Extract	Alessandria Della Rocca Extract
			mg/kg DE		
Gallic acid	111,425.38	197,489.19	12,560.50	136,439.23	114,591.42
Neochlorogenic acid	n.d. ^1^	32.98	n.d.	n.d.	n.d.
Catechin	77.58	116.72	n.d.	25.33	74.74
*p*-Hydroxy benzoic acid	338.70	708.11	36.99	599.73	461.54
*m*-Hydroxy benzoic acid	n.d.	341.39	n.d.	54.74	816.53
Caffeic acid	30.00	114.80	n.d.	27.67	48.73
Vanillic acid	58.80	121.87	n.d.	113.31	93.00
Syringic acid	27.40	47.08	n.d.	99.07	27.40
*p*-Coumaric acid	104.64	294.22	13.82	157.56	163.66
Ferulic acid	18.78	36.23	n.d.	n.d.	n.d.
3,5-Dicaffeoylquinic acid	0.61	0.22	n.d.	n.d.	n.d.
Rutin	83.47	142.93	11.03	170.52	77.47
Isoquercitrin	1564.56	20,342.82	63.75	1152.69	2010.84
Delphinidin-3,5-diglucoside	1347.25	2239.77	69.43	950.77	1785.47
Phloridzin	51.26	222.15	5.00	37.93	117.04
Quercitrin	3022.64	9354.22	185.90	4754.62	5645.66
Myricetin	8.81	103.77	n.d.	2.70	16.77
Kaempferol-3-glucoside	100.32	694.07	27.91	211.91	370.33
Ellagic acid	1603.97	3816.50	444.36	2316.83	2068.79
Quercetin	22.83	648.68	14.09	36.55	67.02
Phloretin	0.07	0.42	n.d.	0.04	0.12
Isorhamnetin	n.d.	0.71	n.d.	n.d.	n.d.
Delphinidin-3-galactoside	22.03	522.75	593.79	35.71	501.42
Cyanidin-3-glucoside	2716.36	17,964.91	20,889.81	1591.86	13,616.93
Pelargonidin-3-glucoside	91.78	196.58	356.10	15.51	127.64
Hyperoside	1291.30	2372.03	2730.72	1033.36	1811.69
Hesperidin	28.88	29.62	31.35	33.08	32.09
Kaempferol	n.d.	101.90	n.d.	1.88	n.d.
Total content	124,037.42	258,056.65	38,034.56	149,862.60	144,526.33

^1^ n.d., not detected.

## Data Availability

The data used to support the findings of this study can be made available by the corresponding author upon request.
